# A Credibility Assessment Plan for an *In Silico* Model that Predicts the Dose–Response Relationship of New Tuberculosis Treatments

**DOI:** 10.1007/s10439-022-03078-w

**Published:** 2022-09-17

**Authors:** Cristina Curreli, Valentina Di Salvatore, Giulia Russo, Francesco Pappalardo, Marco Viceconti

**Affiliations:** 1 Department of Industrial Engineering, Alma Mater Studiorum - University of Bologna, Bologna, Italy; 2 Medical Technology Lab, IRCCS Istituto Ortopedico Rizzoli, Via di Barbiano 1/10, 40136 Bologna, Italy; 3 Department of Drug and Health Sciences, University of Catania, Catania, Italy; 4 Mimesis srl, Catania, Italy

**Keywords:** Tuberculosis, Agent-based model, Drug development, Model credibility, Verification, Validation

## Abstract

Tuberculosis is one of the leading causes of death in several developing countries and a public health emergency of international concern. *In Silico* Trials can be used to support innovation in the context of drug development reducing the duration and the cost of the clinical experimentations, a particularly desirable goal for diseases such as tuberculosis. The agent-based Universal Immune System Simulator was used to develop an *In Silico* Trials environment that can predict the dose–response of new therapeutic vaccines against pulmonary tuberculosis, supporting the optimal design of clinical trials. But before such *in silico* methodology can be used in the evaluation of new treatments, it is mandatory to assess the credibility of this predictive model. This study presents a risk-informed credibility assessment plan inspired by the ASME V&V 40‐2018 technical standard. Based on the selected context of use and regulatory impact of the technology, a detailed risk analysis is described together with the definition of all the verification and validation activities and related acceptability criteria. The work provides an example of the first steps required for the regulatory evaluation of an agent-based model used in the context of drug development.

## Introduction

Tuberculosis (TB) is still a serious health problem in several developing countries, and it is considered today the second leading cause of death worldwide from a single infectious agent, after SARS-CoV-2.^42^ The most recent global TB report published by the World Health Organization^42^ states that about a quarter of the world’s population is affected by TB and about 1.5 million people died in 2020. The number of TB cases varies widely among countries: it is estimated that only eight states of South-East Asia and Africa accounted for almost two thirds of the global TB incidence rate with India the worst affected (26%). Poverty, malnutrition, overcrowding, HIV infection and alcohol consumption are all factors strongly associated with TB.^22^

Tuberculosis, caused by a bacillus named *Mycobacterium tuberculosis* (MTB), typically affects the lungs (Pulmonary TB) and can lead to active and latent forms.^15^ The commonly used first line antibiotic treatments recommended for drug-susceptible TB patients are isoniazid, rifampicin, ethambutol, and pyrazinamide. Second line treatments for drug-resistant and multidrug resistant patients (resistant to both isoniazid and rifampicin) are usually more complex and include long duration antituberculosis therapies that are sometimes prohibitively expensive, especially considering the low average income of many target countries. A new promising strategy that proved to be effective in shortening the treatment duration and in preventing recurrent tuberculosis is a combined approach known as therapeutic vaccination or immunotherapy.^6,14^ Different recent studies demonstrated the possibility to use the combination of TB vaccination and drug treatments to drastically shorten the time to sputum culture conversion and decrease the probability of developing drug-resistant strain^6,43^; however, the clinical experimentation of this therapies is slow and expensive and to date none have entered the market.^42,36^

*In Silico* Trials technologies, used in combination with clinical experimentation, promise to reduce the cost and duration of such trials.^14,29,30,34,40^ The use of computer modelling and simulations is rapidly growing in the pharmaceutical area^41^ and one specific application which is gaining particular interest in the context of drug development and evaluation is the assessment of the optimal drug dose to be used in the clinical study.^25^ The design of these dose–response studies is in fact complex and requires the recruitments of both healthy volunteers and TB patients. Also, because different arms need to be included in the study (e.g., drug-resistant and drug-responsive strains, HIV-positive and HIV-negative patients), only a fairly limited number of doses can be tested over a small group of subjects per each arm.^27^

In the last decades, different modelling and simulation-based approaches have been proposed to test dose–response of new treatments for different applications. Some of the most advanced methods were classified and summarised in References 14, 23. Specifically for TB, the most commonly used computational approach relies on mechanistic and physiologically based pharmacokinetic/pharmacodynamic (PK/PD) models.^4,16,20^ In Reference 20, a computational framework that combines a PK-PD model and a multi-objective optimization approach was presented to identify a set of trade-off optimal dosage regimens for pulmonary TB. Boonpeng *et al*. recently used a population PK model to guide optimal levofloxacin dose regimens for multidrug resistant (MDR) TB therapy^4^; the pharmacokinetics analyses explored the population variability examining different patient characteristics and predicted different dosage regimens using Monte Carlo simulations. A similar mechanism-based PK/PD approach was adopted by Heinrichs *et al*.^16^ that simulated a virtual clinical trial to inform optimal dosage of moxifloxacin and linezolid. Other promising modelling techniques include the use of agent-based multi-scale models, powerful tools to simulate complex phenomena such as the interaction of single cells and molecular entities.^9,10,31^ In 2017, Cicchese *et al*. found that optimal TB antibiotic doses and schedules could be identified using a hybrid model that incorporates Agent-Based models (ABM), PK/PD models and mathematical optimization strategy based on genetic algorithms and radial basis function networks.^9^ More recently, the Universal Immune System Simulator (UISS) has been used as a basis to implement a patient-specific disease progression model for TB (UISS-TB), and a treatment model of the RUTI therapeutic vaccine, indicated for the prevention of active TB in subjects with latent infection.^30^

Although the value of such emerging technologies is widely recognized, one critical aspect that must be properly addressed before using them to support human experimentation in the context of drug development and new market authorization submission is the assessment of the model’s credibility. Recent publications co-authored by officers of major regulatory agencies suggest the possibility to use, also for drug development tools, a recent technical standard proposed to assess the credibility of models for the development of medical devices, the ASME VV-40:2018.^2,24,25,37^ A brief summary of the verification and validation strategy for UISS-TB was introduced in References 11, 25; however, a comprehensive description of all the activities that are needed to evaluate the credibility of the model has never been reported.

The aim of this paper is to present a detailed risk-based credibility assessment plan according to the VV-40 standard of an agent-based *In Silico* trial model to be used in dose selection studies of new therapeutic vaccines against TB. Based on the selected context of use and regulatory impact of the technology, a detailed risk analysis is described together with the definition of all the verification and validation activities and related acceptability criteria. This work provides a general framework that will be submitted as proposed credibility evidence plan for the qualification advice request of the *in silico* methodology to the regulatory authority.

## Materials and Methods

### The Investigational Product and Regulatory Status

Therapeutic vaccines are adjunctive therapies administered along with a specific chemotherapy and used to shorten the TB treatment period, reduce the resistance and the relapse rate. The product formulation and route of administration depend on the specific drug. The vaccine is typically administered along or after the start of the chemotherapy and the duration of the combined therapy can vary significantly (e.g., from 1 to 20 months). Examples of TB therapeutic vaccines are Mycobacterium vaccae and RUTI vaccines. The mechanisms of action of these two vaccines follow the “host-directed-therapy” and “bacilli-directed therapy” respectively, according to the two main design categories described in Reference 7. The first vaccine is made of heat-killed Mycobacterium vaccae that can enhance the host defence against MTB by promoting T-helper (Th) lymphocytes Th1 and suppressing Th2 response. It promotes the generation of CD8 + T cells that increases the production of Interferon-gamma human (IFN-γ), a cytokine with a fundamental role in the body’s immune response when fighting MTB infection since it activates macrophages that kills the intracellular mycobacteria. A phase III clinical study that involved 10,000 participants was conducted in 2013 to assess safety and efficacy of Mycobacterium vaccae. RUTI is a polyantigenic liposomal vaccine made of detoxified, fragmented M. tuberculosis cells (FCMtb), that demonstrated to significantly reduce the bacillary load also thanks to its bactericidal activity. Good safety, tolerability and immunological response was observed in a Phase II study where 96 latent TB infected individuals were evaluated. Immunogenicity, defined as the ability to induce an immune system response was used as an indicator of vaccine efficacy and measured in term of IFN-γ production by blood cells. A Phase IIb clinical trial to investigate the RUTI efficacy in drug sensitive and multidrug-resistant patients has recently started in India.^1^ Other therapeutic vaccine candidates are MVA Multiphasic vac and ID93/GLA-SE which completed preclinical and phase I respectively.

### General Description of the Predictive Model

The computational platform UISS-TB is a specific implementation of the Universal Immune System Simulator that has been recently extended to model the response of the immune system to the pulmonary infection of MTB.^30^ The model is based on an agent-based paradigm and represents the compartment of interest (lung or lymph node) as a bidimensional domain, within which each biological entity (e.g., pathogens, cells or molecular species) can move and interact according to specific cause-effect relationships (e.g., molecular dynamics and string-matching affinity rule). Each agent is mainly characterised by a type, a set of admissible states, a position within the compartment, and a molecular fingerprint (presentation pattern). Stochastic interactions and intrinsic randomness of the complex phenomena are also implemented using three stochastic variables that define the initial distribution of the agents in the space domain, the human leukocyte antigen type and randomisation of the environmental factors (e.g., the effect on the lymphatic flow). Random seed algorithms generators are used to initialize the stochastic variables.^39^

A feature set of 22 model inputs (Table 1) with admissible minimum (*I*_MIN_) and maximum (*I*_MAX_) values identified according to literature data^17,21^ is used to define a possible virtual patient to be considered in the simulation setting. The novel approach from Juárez *et al*.^18^ is then adopted to create the virtual cohort based on the typical values, standard deviation and joint distribution of the target population characteristics.

**Table 1:** Inputs values of the UISS-TB model.

INPUTS	Description	*I*_Min_	*I*_MAX_
Mtb_Vir	Virulence factor	0	1
Mtb_Sputum (CFU/mL)	Bacterial load in the sputum smear	0	10,000
Th1 (cells/*µ*L)	CD4 T cell type 1	0	100
Th2 (cells/*µ*L)	CD4 T cell type 2	0	100
IgG (GMT)	Specific antibody titer	0	512,000
TC (cells/*µ*L)	CD8 T cell	0	1134
IL-1 (pg/mL)	Interleukin 1	0	235
IL-2 (pg/mL)	Interleukin 2	0	894
IL-10 (pg/mL)	Interleukin 10	0	516
IL-12 (pg/mL)	Interleukin 12	0	495
IL17-a (pg/mL)	Interleukin 17A	0	704
IL-23 (pg/mL)	Interleukin 23	0	800
IFN1A (pg/mL)	Interferon alpha-1	0	148.4
IFN1B (pg /mL)	Interferon beta-1b	0	206
IFNG (pg/mL)	Interferon gamma (IFN-γ)	0	49.4
TNF (pg/mL)	Tumor necrosis factor	0	268.2
LXA4 (ng/mL)	Lipoxin A4	0	3
PGE2 (ng /mL)	Prostaglandin E2	0	2.1
VitaminD (ng/mL)	Vitamin D	25	80
Treg (cells /*µ*L)	Regulatory T cells	0	200
Age (years)	Age	10	80
BMI (kg/m^2^)	Body mass index	18	35

The simulation platform is defined based on three different model layers: (i) physiology layer where UISS-TB simulates the physiological response of the human immune system to an infective exposure of MTB; (ii) disease layer that implements the disease mechanism of the pulmonary infection and (iii) the treatment layer that includes the effect of different therapeutic treatments on the development of the pathology.

The modelling application specifically designed to predict the population dose–response relationship for the therapeutic vaccine is called UISS-TB-DR and includes all the three model layers, considering both the first line antibiotic therapy and the second line vaccine mechanism of action in the treatment model. The model simulates the infection of a representative portion of the pulmonary compartment by MTB and considers the pathogen with genomic polymorphisms, including their debris, macrophages, neutrophils, dendritic cells, regulatory T cells, B cells, and CD4+ and CD8+ Th cells. Each treatment is modelled on the basis of the known mechanism of action, the treatment plan, and the patient-specific drug susceptibility/resistance test. To implement a vaccine mechanism of action and predict its effects in terms of stimulated immune response against MTB and the provoked tuberculosis disease, UISS-TB-DR needs to consider the vaccine formulation, dosage, biochemical properties (i.e., half-life) and the specific interactions of all the components that constitute the vaccine with the host simulated immune system. All these properties are retrieved from preclinical studies. The methods used to translate dose results in humans are based on allometric scaling (e.g., normalizing dose-to-body surface area). Five steps are usually performed to extract data from preclinical studies that include fundamental toxicology trials: (i) determination of no observed adverse effect levels (NOAEL) in animal toxicity studies; (ii) the conversion of NOEL to human equivalent dose; (iii) selection of appropriate animal species; (iv) apply safety factor; (v) consider pharmacologically active dose.^26^

### Validation Data

The observed data used for the validation study (“Validation” section) are described in the work published by Nell *et al*.,^27^ In the phase II clinical trial, carried out by three South African sites (Bloemfontein, George, and Port Elizabeth) from July 2010 to April 2011, three different doses (5, 25 and 50 *µ*g) of RUTI vaccine are evaluated in term of safety, tolerability and immunogenicity compared to placebo in subjects with latent tuberculosis infection. The study involved 96 patients without evidence of active TB (men and women 18–50 years of age including 47 HIV-positive and 48 HIV-negative). Each subject was randomized to receive one of the four treatments: placebo, 5, 25 and 50 *µ*g of RUTI vaccine at days 28 and 56 after completion of one month of isoniazid (INH, one tablet of 300 mg/day). Tolerability and safety were mostly qualitatively evaluated (e.g., pain, swelling, induration, functional limitation, vital sign and adverse events assessment) while immunogenicity was accurately measured in a time frame of 63 days. Cellular mediated immunity was assessed using ELISPOT and ELISA techniques and IFN-γ Spot Forming Units were measured with WHO and TIGRA (T-SPOT TB) assays. Humoral responses were also studied. Five time points were identified at day 0, 28 (pre-1st dose), 35, 56 (pre-2nd dose) and 63.

### Risk-Informed Model Credibility

#### Question of Interest and Context of Use (CoU)

The scientific question of interest to be addressed is “what is the most immunogenic dose of the new therapeutic vaccine to be used in patients affected by tuberculosis?”.

This is a critical issue is the context of dose selection studies since immunogenicity, as indicator of vaccine efficacy, contributes to the identification of the optimal vaccine dose together with safety and tolerability evidence. Also, the highest dose does not necessarily produce the highest immune response which can be non-linear and might vary depending on the time considered after the drug administration. The UISS-TB-DR model will be used to support the decision about the most immunogenic dose of the new therapeutic vaccine against TB and inform phase II dose selection studies by predicting the human immune system response representative of a real population in terms of immunogenicity. Based on the results of preclinical experiments and phase I clinical studies (i.e., minimum effective and maximum tolerable doses), simulations are run taking into account patients specific characteristics. The model results in term of IFN-γ concentration will be used to extract an average dose–response curve and to identify the dose that produces the highest immunogenic response. The characteristics of the real patients will be used to build an “extended” virtual cohort that, in addition to the treatment formulations and route of administration, will be considered as inputs to the simulation platform. The levels of IFN-γ predicted by the model will be used as immunogenicity response biomarker to suggest the appropriate dose of the treatment for which the marketing authorisation is requested to be tested on a real cohort of patients during phase II dose selection studies. The process we propose uses UISS-TB-DR to predict the response over a virtual population much larger than the one normally used in such experimental studies. The results obtained with the *in silico* model will be used to confirm and support evidence from phase I clinical experiments and inform phase II dose selection studies.

#### Regulatory Impact

Considering the definitions presented in References 17, 20, and 21 where the concept of regulatory impact is related to how regulators weigh the importance of models compared to alternative methods to address the final regulatory question, the regulatory impact of the developed modelling framework for the proposed CoU is low. An iterative and stepwise approach process will be adopted for the qualification advice request to the regulatory agency. As a first step, the computational methodology is proposed to confirm the available clinical evidence and support the selection of the most immunogenic dose of the new therapeutic vaccine obtained during phase II dose selection studies. However, it is important to notice that the final intended regulatory use of the UISS-TB-DR model is to reduce the human experimentation required to choose the optimal immunogenic dose of a new therapeutic vaccine. Based on the taxonomy presented in References 40, we position our application in the general category “reduce Clinical Human Experiments” with the final aim to optimize and accelerate TB drug development reducing the number of humans involved in the experiment, and its cost/duration. Once the methodology will be tested and validated for different therapeutic treatments and case studies, the modelling and simulation framework can be considered an alternative method that can be used to assess immunogenicity in the dose–response studies, replace the usual evidence and drastically reduce the number of patients enrolled in the clinical trials.

#### Model Risk Assessment

The model risk assessment defined in the ASME VV-40 standard is based on the combination of model influence and decision consequence. The definition of model influence is somehow related to the concept of regulatory impact described in the previous section; but it better stresses the contribution of the model on the final decision versus other available evidence used to address the question of interest.

For the CoU previously described, the *model influence* can be considered *low* because the results of the computational model represent a minor factor in the final decision. The most immunogenic dose will be mainly selected based on confirmatory clinical studies and other evidences about safety and tolerability.

The *Decision* consequence is *medium*: an incorrect decision could have a moderate impact for the patient especially if unfavourable benefit-risk balance is estimated. In case the selected dose is moderately sub-optimal (a grossly sub-optimal dose would be detected by the confirmatory experiment), the treatment might result to be slightly less effective, but no serious adverse effects related to safety and tolerability would be expected (usually carefully tested in preclinical and phase I studies).

**Figure 1:**
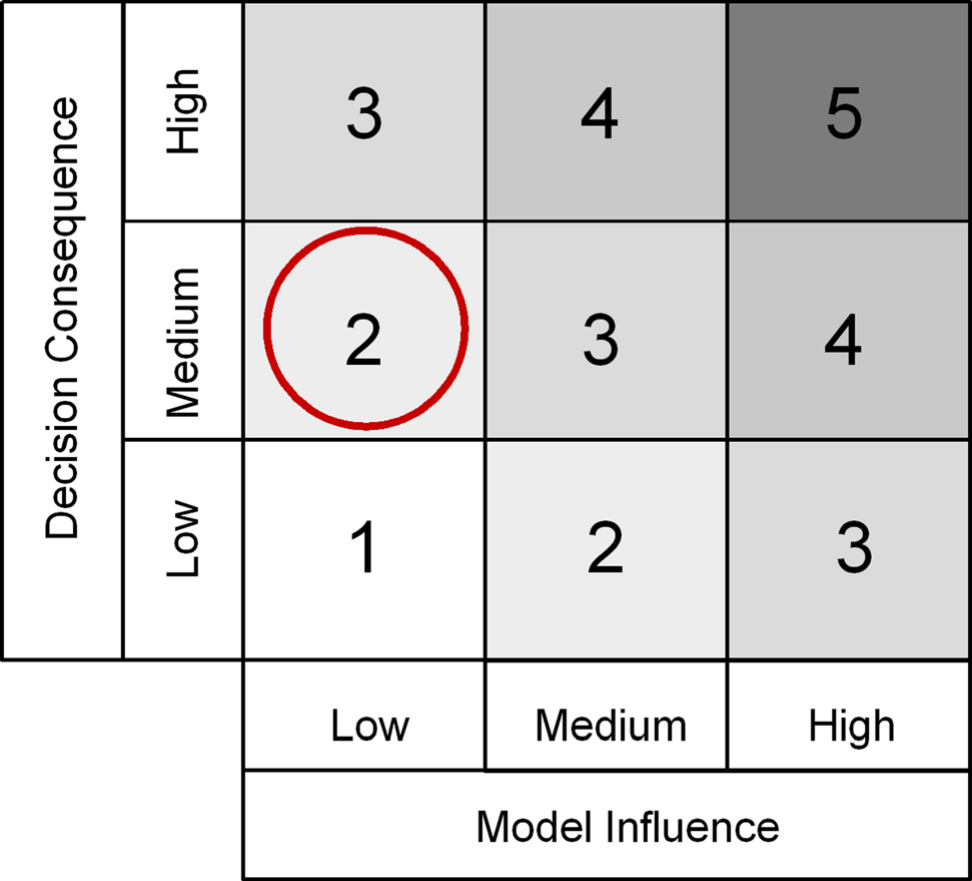
Model risk map indicating model influence and decision consequence for the CoU.

The credibility assessment plan is thus designed assuming the risk associated with the use of UISS-TB-DR for this specific context of use as medium–low (Fig. 1).

## Results

### Credibility Activities Plan and Goals

Following the ASME VV-40:2018 standard, the level of risk and regulatory impact guide the definition of the credibility assessment plan. The activities selected for UISS-TB-DR model are detailed in Table 2, together with the relative credibility factors and goal, also defined consistently with the VV-40 standard.

**Table 2:** Verification, validation and applicability activities with their credibility factors. Outline of the credibility goal is also reported.

Activities		Credibility factors	Credibility goals
Verification	Code	Software quality assurance	Low
		Numerical code verification	
	Calculation	Existence, uniqueness and use error	Medium
		Input/output exploration	
		Consistency and sample size	
Validation	Computational model	Model form	Medium
		Model inputs	
	Comparator	Test samples	Low
		Test conditions	
	Assessment	Equivalency of input parameters	Low
		Output comparison	
Applicability		Relevance of the quantity of interest	Medium
		Relevance of the validation activities	

According to the CoU, the verification and validation activities are defined considering as quantity of interest (QoI), the concentration of IFN-γ that is used to test immunogenicity and suggest the appropriate dose of the treatment.

### Verification

#### Code Verification

*Credibility goal*: *Low*. *Little SQA and numerical code verification procedures are specified*

Software quality assurance procedures include quality metrics tracking such as correctness and reproducibility. A specific benchmark test is replicated to ensure that the software is functioning correctly and produces previously established results. In particular, two generic challenges with extracellular bacteria have been performed at month 0 and at month 3, in order to assess the antibodies-mediated immune response directed against the generic bacterium one week after the first challenge and one week after the second one. The comparison between the two antibody levels showed an increase after the second challenge which should be almost doubled compared to the previous phase.^32,35^ The GitHub platform is also used to enforce quality control, usability, portability and versioning control (e.g., code review and debugging).

#### Calculation Verification

*Credibility goal: Medium*. *Problem specific calculation verification study is conducted and key inputs/outputs are verified by internal peer review*.

Calculation verification is performed based on the methodology described in a recently published paper^11^ that is specifically designed for agent-based models and distinguishes between deterministic and stochastic model verification activities. The first group includes all the tests performed considering fixed values for the stochastic parameter values (Existence, Uniqueness and Use Error; Input/Output exploration) while the second aims at investigating the effect of the randomization factors and includes: Consistency and sample size determination. The verification activities with the identified quality metrics and requirements for model acceptability are described below.

##### Existence, Uniqueness and User Error

It aims at verifying basic essential properties such as existence and uniqueness of the solution. Key inputs and outputs are also verified to ensure that no errors occur in the simulations due to the practitioner (e.g., typographical errors).

##### I/O Exploration

The goal of this activity, also called parameter sweep analysis, is to verify that the model is not numerically ill-conditioned and behaves reliably for all the input value combinations. The entire input sets space is sampled uniformly using a Design of Experiment (DoE) approach and considering the possible minimum and maximum values for all the variables (Table 1). A total of 100 simulations are then run for all the admissible drug doses to verify that any combinations of inputs do not lead to unexpected trends of the model’s corresponding outputs (e.g., concentration of IFN-γ values outside the “biological acceptable” concentration range). The global variation effect of the input parameters on the output results is also quantified through coefficient of variation *C* defined as the ratio between the standard deviation and the mean for each output. This value should not be greater than typical variability results (e.g., around 70%) obtained from clinical studies across multicentre trials and continents.^3^

##### Consistency and Sample Size Determination

A total of 1000 simulations varying the value of the stochastic parameters and setting all the other inputs to their mean value are run and studied in terms of statistical consistency. In particular, a graphical check on the histograms and the Kullback–Leibler (KL) divergence measure^38^ is used to characterise the shape of the sample distributions for the QoI and their fit to gaussian and Student-t distribution. Also, typical descriptive statistics (i.e., mean, standard deviation and coefficient of variation) are computed. We assume the model is stochastically consistent if the ratio between the divergence of the Student-t (KLs) and the Gaussian (KL_G_) are in the range [0.8–1.2]. The number of simulation runs (number of samples obtained with different values for the stochastic variables) required to have a statistical significance is computed assessing the variability of the measurements and evaluating the coefficient of variation rate as described in Reference 11. A sample size of 600 simulation runs is considered acceptable assuming the computational cost as a major constraint. The time required to run the entire simulation with an Intel Core i7-9700 at 3 GHz and 32 GB RAM for one single digital patient is about one minute.

### Validation

Validation aims at assessing the degree to which the computational model is an appropriate representation of the reality of interest. The validity of some model form and input assumptions are tested in the activities related to *Model Form* and *Model Input* with uncertainty quantification analyses. Then, credibility factors related to the comparator data samples (*Test samples*) and conditions (*Test conditions*) are evaluated to examine the rigor of the comparison in terms of number and characteristics of test samples used and measurements of the test conditions. Finally, model assessment is evaluated analysing the *Equivalency of Input Parameters* and *Output Comparison.*

#### Computational Model

*Credibility goal: Medium. Influence of expected key model form assumption and uncertainties on expected key inputs is explored*.

I. Model Form

The key modelling form assumptions are related to the definition of the computational domain used for the simulations (i.e., 1 *μ*L of peripheral blood sample) and the time frame used to expose the virtual patient to the MTB challenge (i.e., 730 days before the antibiotic treatment starts). The influence of the model form assumptions was explored by quantifying their impact on the output results. The simulations are repeated considering a blood volume of 10 and 100 *μ*L and a time interval of 730 ± 10 days for the MTB exposure challenge. The model form assumptions were assumed to not significantly impact the decision related to the CoU for percentage differences of the IFN-γ concentration inferior to 10%.

II. Model Inputs

Uncertainty quantification analyses are conducted on the key model inputs: MTB_Vir, MTB_Sputum, VitaminD, Treg, Age, and BMI. All these input features, describing biological and patho-physiological parameters, are fundamental in TB infection dynamics and normally measured clinically. A novel statistical Bayesian approach described in Reference 19 is used to define the value ranges accounting for the uncertainty in the augmented clinical trial. Considering a reference input vector of features that identify one virtual patient, the analyses are performed perturbing the six inputs one at a time from their minimum to their maximum value. A variation range on the IFN-γ concentration less than 10% is considered as acceptable.

#### Comparator

*Credibility goal: Low. One in vivo experimental study reported in the literature is considered for the comparison that includes limited information on the patient characteristics and test conditions*.

Test samples and test conditions of the comparator data (“Validation Data” section) are identified and used to build the *in silico* clinical trial. Three different virtual cohorts (for the same three treatment RUTI vaccine doses 5, 25 and 50 *µ*g) are built considering the mean value and standard deviation of the parameters representing the main characteristics of the HIV-negative latent tuberculosis patients enrolled in the clinical study.^27^ A total number of 12 patients were included in each arm of the clinical trial, while for the augmented *in silico* study, 100 virtual patients were modelled in each of the three virtual cohorts following the methodology presented in Reference 18. Test conditions are characterised in terms of route of administration and duration of the treatments (i.e., the virtual patients are exposed to an infective MTB challenge and then treated with the RUTI vaccine after completion of one month of INH as reported in the clinical study).

#### Assessment

*Credibility goal: Low. Visual comparison and quantitative agreement measurement is performed on one single output quantity*.

I. Equivalency of Input Parameters

The type and range of the input parameters are similar but not equivalent. From the comparator data, the mean value and standard deviation of two patient features can be extracted and used as model inputs (i.e., age and weight) while the other variables listed in Table 1, were defined based on probability distributions data presented in the literature.^19^

II. Output Comparison

A single simulation output (i.e., concentration of IFN-γ expressed in SFU/ 0.25 × 10^6^ cells) is compared with the observed results of the clinical study for three different time points (day 35, 56, 63). The standard numerical and graphical analysis described in the EMA guidance on reporting pop-PK is considered appropriate.^13^ Goodness of fit plot with quantitative agreement measurements (e.g., percentage difference between computational results and the *in vivo* data obtained from the clinical study) is considered. Because the comparison is not at the individual level but a statistical agreement assessment is performed, the model credibility evidence falls in the category defined as population based evidence.^5^ In both comparator and computational model results, the most immunogenic dose of the RUTI therapeutic vaccine is considered the one that produces a higher concentration of IFN-γ in the observed population.

#### Other Validation Evidences

General non-CoU related evidence is also produced to support model credibility. In particular, evidence that demonstrates that the model reproduces phenomena that are known to occur in humans are reported considering qualitative experimental observations described in the literature at two different layers: physiological and disease layer. Visual comparison is performed between the predicted and expected trend of the quantities and behaviours briefly presented below.

I. Physiological Layer

Different UISS-TB output predictions regarding tuberculosis immunological hallmarks during the early infection phase are considered for the physiological layer.^28^ These include: (i) innate early host immune response to MTB infection; (ii) adaptive T cellular immune response mounted against MTB infection; (iii) dynamics of the typical CD4 + Th1 and Th17 cytokines signature; (iv) dynamics of IL-10, TNF-α, type I interferons, LXA4 and PGE2; and (v) dynamics of Mtb viable bacilli and specific IgM, IgG and IgA anti-Mtb.

II. Disease Layer

Model credibility evidence at the disease layer aims to demonstrate that UISS-TB reproduces the natural history of pulmonary tuberculosis infection.^33^ Model predictions are compared in term of: (i) rate of mortality over a population of untreated subjects exposed to the MTB infection; (ii) rate of patients with latent MTB infection that eventually develop the active form of disease over a period of time; and (iii) changes in representative lymphocytes populations in the transition from latent to the active form.

### Applicability

*Credibility goal: Medium. The quantity of interest is relevant to support the use of the model for the intended use. A partial overlap between the validation points and the CoU is observed.*

The applicability analysis is performed considering the relevance of both the QoI and the validation activities for the CoU. The simulation output selected for the validation study is the concentration of IFN-γ which is identical to the quantity used as an accepted biomarker of the immunogenicity response. The identified quantity of interest is thus fully relevant to the CoU. However, a partial overlap between the context of use and the validation points can be observed: only one therapeutic vaccine is considered in the comparator study with test conditions and samples that are limited to one clinical trial.

## Discussion

The aim of this study was to develop a detailed risk informed model credibility plan that can be used in the qualification advice request submission to support the overall credibility of the computational model for the CoU. Based on the specific scope and role of the computational model used to address the question of interest and the established regulatory impact, a model risk analysis has been performed and used to define the credibility factors and goals. Specific factors for calculation verification of agent-based models have been selected while typical validation activities related to the computational model, comparator and assessment have been considered. According to the identified regulatory impact and model risk, a low-medium level of investigation into each factor is considered acceptable and the credibility evidence, if the credibility goals are achieved, are expected to be sufficient to support using the model for the CoU. The QoI considered for the model validation is applicable to the question of interest and CoU: the concentration of IFN-γ extracted from the computational platform is in fact typically used to test immunogenicity in dose selection studies. Also, the validation activities are performed using *in vivo* experimental data reported in the literature that are relevant to the CoU; however the available dataset is related to only one type of therapeutic vaccine and includes limited information on the patient characteristics and test conditions (e.g., clinical protocols followed). For this reason, the credibility level assigned to the validation factors, especially for the comparator and assessment activities, is low. As already mentioned (“Risk-Informed Model Credibility” section), the UISS-TB-DR model will be used to inform the decision about the most immunogenic therapeutic vaccine dose using an augmented virtual cohort that will allow a more extensive evaluation of the treatment effect; however, final decision will be taken based on the results of the clinical studies where the different vaccine doses would be tested on a real cohort of patients. More extensive validation studies will be then conducted when the computational model will be proposed for its final regulatory use: reduce clinical human experiments and/or the duration of the clinical experiments and accelerate TB drug development.

An iterative and stepwise approach process is proposed for the qualification advice request to the regulatory agency. A first intermediate step is presented in this work as a proof-of-concept test to consolidate the main blocks of the model credibility framework and the definition of the model acceptability criteria. Among the main challenges reported by sponsors/developers are in fact lack of guidance for the definition of the methodology that can be used to establish credibility plans that strongly depend on modelling approach and applications.^25^ Both the Food and Drug Administration and the European Medicines Agency suggest adopting a step-by-step approach and considering early interaction between developers and regulatory authorities to facilitate the evaluation of the proposed rationale for credibility and prevent the definition of inappropriate data generation plan.^8^ A prospective adequacy assessment of the credibility plan stating if and why the evidence and goals are considered sufficient for the proposed CoU model is also recommended before performing the V&V activities.

The guidelines reported in the recently published FDA draft document^5^ are considered in this study to identify and categorise some of the proposed credibility evidence. Non-CoU related validation studies are also included in the plan to provide a more in-depth understanding of the predictive capability of the computational model demonstrating the ability of the model to reproduce general pathophysiological behaviours in TB.

This study presents the first steps required for the regulatory evaluation of UISS-TB-DR. A credibility plan inspired by the ASME V&V40 standard has been described and applied for the first time to an *in silico* agent based model that will be used to support innovation in the context of drug development against tuberculosis.

## Abbreviations

ABM: Agent-based model
CoU: Context of use
FCMtb: Fragmented mycobacterium tuberculosis cell
IFN-γ: Interferon-gamma human
KL: Kullback–Leibler divergence measure
MDR: Multidrug resistant
MTB: Mycobacterium tuberculosis
PD: Pharmacodynamic
PK: Pharmacokinetic
QoI: Quantity of interest
TB: Tuberculosis
Th: T-helper cell
UISS: Universal Immune System Simulator
UISS-TB-DR: Universal Immune System Simulator designed to predict the population dose–response relationship for therapeutic vaccine against tubercolosis
